# A Non-coding HES1 Variant Predisposes Children to Congenital Heart Disease in Chinese Population

**DOI:** 10.3389/fcell.2021.631942

**Published:** 2021-01-28

**Authors:** Yangliu Song, Weicheng Chen, Zitong Huang, Guixiang Tian, Mengru Li, Zhengshan Zhao, Zhiyu Feng, Feizhen Wu, Maoxiang Qian, Xiaojing Ma, Wei Sheng, Guoying Huang

**Affiliations:** ^1^Institutes of Biomedical Sciences, and Children's Hospital of Fudan University, Shanghai, China; ^2^Shanghai Key Laboratory of Birth Defects, Shanghai, China

**Keywords:** congenital heart disease, HES1, non-coding variant, transposition of the great artery, RXRA

## Abstract

**Background:** As a key component in the NOTCH signaling pathway, HES1 plays an important role in vertebrate heart development. Variants in the HES1 coding sequence are known to be associated with congenital heart disease (CHD). However, little is known about HES1 non-coding sequence variants and their association with the risk of developing CHD.

**Method and Results:** We initially analyzed the non-coding sequence of the HES1 gene in 12 unrelated CHD families by direct sequencing and identified a previously unreported promoter region variant (NM_005524.4: c.−1279−1278 insAC, rs148941464) in the HES1 gene in four CHD families. The homozygous variant in patients was inherited from carrier parents with normal phenotypes, indicating a likely recessive genetic model. Given that the HES1 gene is predicted to be likely to exhibit haploinsufficiency (%HI: 11.44), we hypothesized that the HES1 homozygous variant is a genetic risk factor underlying CHD. We then carried out sequencing of this HES1 variant in 629 sporadic non-syndromic CHD cases and 696 healthy controls and performed association analysis. Interestingly, we observed a significant association of the homozygous HES1 promoter variant with CHD (18.92% of cases vs. 9.91% of controls; OR: 2.291, 95% CI: 1.637-3.207, *p* = 9.72 × 10^−7^). No significant association with CHD was observed for the HES1 promoter heterozygous variant (*p* > 0.05). However, association analysis tests of the HES1 homozygous variant with each subtype of CHD revealed that this homozygous variant was strongly associated with transposition of the great arteries (TGA) (OR: 3.726, 95% CI: 1.745-7.956, *p* = 0.0003). Moreover, the prevalence of HES1 homozygous variants in CHD patients with TGA (27.66%) was significantly higher than that in patients with other CHD subtypes or controls. Similar results were observed in a replication group of TGA (*n* = 64). Functional studies demonstrated that the homozygous variant in the HES1 promoter can disrupt its ability to bind RXRA, an inhibitory transcription factor, which results in abnormally high expression of the HES1 gene, indicating that this variant harbors gain-of-function effects.

**Conclusions:** Our findings reveal that the non-coding homozygous variant in the HES1 promoter has a gain-of-function effect and is associated with an increased risk of CHD development, especially the severe TGA subtype.

## Introduction

Congenital heart defects (CHDs) are the most common birth defects caused by the disturbance of heart formation during embryonic development, with a prevalence of 8.98‰ among live births in China (Zhao et al., 2019). CHD phenotypes are complex and range from single, localized defects (for example, VSD and ASD) to more complex structural malformations (for example, TGA and TOF). Among these CHD subtypes, CHDs with complex structural malformations, such as TGA, are considered the most severe CHD subtypes; these are the leading cause of newborn deaths and also the cause in ~10% of cases of fetal demise (Jorgensen et al., 2014; Liu et al., 2020).

The etiology of CHD is complex and includes both genetic and non-genetic risk factors. Although non-genetic risk factors, such as environmental teratogenic factors, maternal teratogenic factors, and maternal infections (Abdulla, 1998), have been proven to be associated with CHD, an increasing number of studies in human and animal models have also identified genetic etiologies underlying CHD. Approximately 400 genes are estimated to be associated with CHD pathogenesis, including genes encoding transcription factors, cell signaling molecules, and structural proteins that are responsible for heart development (Lage et al., 2012; Li et al., 2015). Pathogenic variants in the coding exons of these genes can perturb the structure and function of the heart by influencing cell type specification, differentiation and pattern formation, leading to the occurrence of CHD.

NOTCH signaling is a highly conserved pathway that has been well-characterized in regulating cell proliferation, differentiation, and apoptosis in organogenesis in almost every tissue and organ. During heart development, the NOTCH signaling pathway is involved in the formation of the atrioventricular ducts, valves, outflow tracts, and trabeculae, and it plays an important role in maintaining the normal development of the second heart field (SHF) (de la Pompa and Epstein, 2012).

Previous studies have shown that mutations and abnormal expression of candidate genes in the NOTCH signaling pathway will lead to many types of CHD (Luxán et al., 2016). Garg et al. found NOTCH1 truncation mutations in a CHD family, and the carriers showed a variety of aortic and cardiac structural abnormalities, such as TOF, DORV, and PS (Garg et al., 2005). The association between NOTCH1 mutation or copy number variation and CHD has also been confirmed in a series of subsequent studies (Greenway et al., 2009; Wang et al., 2011). Whole-exome sequencing (WES) was performed to assess the incidence of unique, deleterious variants in non-syndromic TOF cases and proved that the NOTCH1 gene is the most frequent site of genetic variants and that the other NOTCH pathway genes tested are not a major cause of TOF in our cohort (Page et al., 2019).

As a key component in the NOTCH signaling pathway, HES1 plays an important role in vertebrate heart development (van Bueren et al., 2010). A previous study found that HES1 is expressed in the SHF and that this expression is necessary for the occurrence of cardiac outflow tracts (Rochais et al., 2009). Abnormal HES1 expression was observed in a variety of CHD types, indicating strong biological sensitivity to HES1 dosage, which has been confirmed in multiple animal models (Yuan et al., 2017; Wu et al., 2018; Zhang et al., 2018). Interestingly, our previous study found abnormal expression of HES1 in the myocardial tissues of TOF patients with no pathogenic variant in the coding region of HES1. Because genome-wide methylation sequencing data showed no differential methylation in the HES1 promoter region in TOF patients, we hypothesized that HES1 non-coding sequence variants associated with the risk of CHD development might be present.

In the current study, we analyzed the non-coding sequence of the HES1 gene by direct sequencing and identified an unreported promoter region variant that showed *in vitro* gain-of-function effects as a homozygous variant and was strongly associated with an increased risk of CHD development, especially the severe subtype TGA.

## Materials and Methods

### Study Subjects

A total of 629 non-syndromic CHD patients were recruited from the Children's Hospital of Fudan University as the cases, and 696 unaffected healthy persons were recruited as the controls (Table 1). Blood samples were collected and stored from volunteers from a racially diverse population, including many residents of the Yangtze River Delta region of China.

**Table 1:** Clinical characteristics of the CHD patient cohort.

	**Combined sample**
	***N* = 629**
**Basic characteristic**	
Age, years (mean ± SD)	2.33 (±3.23)
Gender	
Male, *n* (%)	357 (56.76)
Female, *n* (%)	272 (43.24)
**Subtype phenotype**	
Ventricular septal defect	293 (46.58)
Atrial septal defect	101 (16.06)
VSD and ASD	31 (4.93)
Tetralogy of Fallot	96 (15.26)
Transposition of the great arteries	40 (6.36)
Pulmonary atresia	37 (5.88)
Right double outlet right ventricle	13 (2.07)
Other complex cardiac malformations	18 (2.86)

Heart tissues were collected from 26 CHD patients undergoing surgery at the Children's Hospital of Fudan University. All CHD patients received general anesthesia and extracorporeal circulation (CPB) during the procedures. Five normal heart tissue samples were collected from children who died in accidents, such as traffic accidents, and underwent autopsy in the Department of Forensic Medicine of Fudan University. The relevant characteristics of all tissue samples are listed in Supplementary Table 1.

The study was approved by the Fudan University Children's Hospital Ethics Committee (CHFU) following the Declaration of Helsinki, and the children's guardians signed the informed consent form.

### Genotyping

Genomic DNA was extracted from the peripheral blood of CHD patients and normal controls by a QIAamp DNA Blood Mini kit (Qiagen, Germany). The concentration and purity of the DNA were measured using a NanoDrop One spectrophotometer (Thermo Scientific, Wilmington, USA). The ECR sequence of the HES1 promoter was identified based on the genomic sequence of the human HES1 gene (NM_005524.4) and subsequently amplified by PCR using specific primer pairs (HES1-detect-F: GAAAACCCCAAGCCCGAAAG and HES1-detect-R: ACCCCGTCTTTCAGAAATTCC). Sequencing was performed using BigDye Terminator v3.0 reagents on a 3730 DNA Analyzer (Applied Biosystems, USA) at Shanghai JieLi Biological Co., Ltd. Samples were genotyped for the HES1 promoter variant using Mutation Surveyor software (SoftGenetics, USA). All primers used are summarized in Supplementary Table 2.

### Immunohistochemistry

An anti-HES1 (1:200, 71559, Abcam, Cambridge, UK) antibody was used for immunostaining. Paraffin-fixed heart tissues were first dewaxed in xylene and then submitted to antigen retrieval in citrate buffer (10 mM pH 6.0) for 10 min with boiling in an autoclave. In addition, endogenous peroxidase was inactivated by 10 min incubation in 3% hydrogen peroxide. After the sections were processed as described above, according to the product instructions, an IHC Detection kit (Gene Tech, Shanghai, China) was used for antigen incubation and staining. Sections were incubated with anti-HES1 at 4°C overnight, incubated with secondary antibodies for 1 h at 37°C, and finally stained with DAB chromogen. The Leica DVM2500 digital microscope system (Leica, Wetzlar, Germany) was used to take pictures of the stained sections, and then the area ratio and average integrated optical density (OD) of the positively stained areas were measured using Image-Pro Plus software. The differences in protein expression between controls and patients were analyzed with the Mann-Whitney U test by SPSS (version 22.0, IBM, Armonk, NY).

### RNA Extraction and RT-PCR

TRIzol Reagent (Invitrogen, CA, USA) was used to extract RNA from heart tissue. The clinical information associated with each tissue is listed in Supplementary Table 2. The quality and integrity of the extracted RNA were assessed before use. According to the instructions of the TaKaRa reverse transcription kit, a primer mix containing oligo(dT) primer and random primer was used as the reverse transcription primer to reverse transcribe RNA to generate cDNA. RT-PCR was performed in triplicate on an ABI 7900 system (Applied Biosystems, Foster City, CA, USA) using SYBR Premix (TaKaRa, Japan). GAPDH was used as the internal reference gene for standardization, and the mRNA expression level of the target gene in the sample was calculated by the 2^−ΔΔ^Ct method. The primer sequences used in the RT-PCR analyses are listed in Supplementary Table 2. The mRNA differences among the three genotypes were analyzed with the Mann-Whitney U test.

### Plasmid Construction

To generate a reporter plasmid (HES1-ECR-Luc), an 850 bp *HES1* (ENSG00000114315) promoter sequence (evolutionarily conserved region, ECR) was amplified by PCR using human genomic DNA as a template. The primer pairs used for PCR are shown in Supplementary Table 2. The amplified fragment was inserted into a pGL3 promoter vector (Promega, Madison, WI) digested with KpnI/XhoI and KpnI/NheI. To perform site-directed mutagenesis of the *HES1* promoter, we used a KOD -Plus- Mutagenesis kit (Toyobo C., Ltd. Life Science Department Osaka Japan). With the HES1-ECR-Luc plasmid as a template, we used specific primers amplified by overlapping PCR to construct the HES1-ECRm-Luc plasmid containing the promoter variant. The plasmid sequences were then verified through Sanger sequencing (Shanghai JieLi, Shanghai, China).

The RXRA (NM_002957) pcDNA3.1-Myc-His expression plasmid was purchased from Changsha Youbao Biotechnology Co., Ltd., and RXRA siRNA was purchased from Shanghai Invitrogen.

### Cell Culture and Luciferase Reporter Assay

Cell lines (293T, NIH3T3, HeLa, HL1, AC16, and H9C2) were adherently cultured in high-glucose Dulbecco's modified Eagle medium containing 10% fetal bovine serum and Pen/Strep (Gibco) incubated at 37°C in 5% CO_2_.

The pGL3-ECR construct was cotransfected with a Renilla luciferase reporter (pRL-TK, Promega) using Lipofectamine 3000 (Invitrogen). The luciferase activity was detected using a dual-luciferase reporter gene kit (Promega). The pGL3 promoter plasmid group was set as the control group, with the detected Renilla luciferase activity serving as the standardized internal reference. Three replicates were set up for each experimental group, and three independent experiments were performed.

### Generation of HES1 Promoter Mutant Cell Lines

The minimal off-target CRISPR dual nickase (Cas9-D10A) was designed at http://crispr.mit.edu/ to flank the targeted mutation at−1279insAC in the human HES1 promoter and cloned as described at http://www.genome-engineering.org/crispr/ HeLa cells were cotransfected with the most efficient selected gRNA pair and a repair template encoding the mutant HES1 promoter sequence using Lipofectamine 3000 (Invitrogen). The transfected cells were gently plated onto 100-mm plates, and selection was initiated 24 h later with medium containing 0.20 μg/ml puromycin. Single puromycin-resistant colonies were picked in 2–3 weeks and verified using PCR and Sanger sequencing. Correctly targeted clones were picked into six-well plates and expanded. The sequences of the gRNAs used for genomic editing are shown in Supplementary Table 2.

### Zebrafish Transgene Assay

Animal care and experimental protocols were approved by the Department of Laboratory Animal Science of Fudan University. All studies complied with the guidelines of Directive 2010/63/EU of the European Parliament on the protection of animals used for scientific purposes or the NIH Guide for the Care and Use of Laboratory Animals. All zebrafish experiments were performed on embryos younger than 72 hpf, and euthanasia was performed by rapid freezing followed by maceration.

Using human genomic DNA containing wild-type genotypes and homozygous variant variants as templates, the ECR fragment of HES1 was amplified by PCR. The primer pairs used are shown in Supplementary Table 2. The product fragments were individually inserted into the pTol2-E1b-GFP (Ritter et al., 2010) plasmid backbone at the BglII/XhoI site to construct recombinant plasmids containing wild-type and mutant ECR fragments. This plasmid backbone contains an E1b promoter driving GFP expression and two Tol2 transposon sites. The mMessage mMachine Sp6 kit (Ambion) was used to transcribe the Tol2 transposase mRNA *in vitro*.

Then, 20 ng/μl recombinant plasmid (pCNE-ECR or pCNE-ECRm) and 50 ng/μl Tol2 transposase mRNA were comicroinjected into naturally mated fertilized wild-type zebrafish eggs at the single- and two-cell stages. The injected embryos were incubated at room temperature, and then the Leica DFC310 FX microscope system (Leica, Wetzlar, Germany) was used to observe GFP expression and to take pictures at the appropriate timepoints to detect the activity of the E1b promoter.

### Electrophoretic Mobility Shift Assay

HeLa cells were transfected with p-RXRA for 48 h and harvested in our laboratory. The nuclear protein contents were extracted using NE-PER Nuclear and Cytoplasmic Extraction Reagents (78835, Thermo Scientific), and their concentrations were measured using the BCA protein assay kit (Takara). Biotin-labeled and unlabeled double-stranded oligonucleotide probes were synthesized at Shanghai General Biotechnology Co., Ltd. The sequences of the wild-type and mutant probes are shown in Supplementary Table 3.

The LightShift Chemiluminescent EMSA kit (20148, Thermo Scientific) was used to assay the binding of probes and protein extracts *in vitro*. To prepare a binding buffer, 1 μg/μl poly(dI·dC), 50% glycerol, 1% NP-40, 100 mM MgCl_2_, and ddH_2_O were added to the 10X binding buffer. Ten micrograms of protein extracts and biotin-labeled probes were incubated in binding buffer for 20 min at room temperature. In the competition group, protein extracts and 200-fold molar unlabeled probes were incubated for 15 min in advance. Samples of the reaction solution were loaded into 6% polyacrylamide gels, and electrophoresis was performed at 100 V in 0.5X TBE for ~45 min. Then, the gel contents were transferred to a nylon membrane at 384 mA for 50 min. Detection was performed using a streptavidin-horseradish peroxidase conjugate, and the membranes were photographed using a Fujifilm Las3000 Luminescent Image Analyzer (Fuji Life Sciences, Tokyo, Japan).

### Shift-Western Blotting

To identify the protein components in the protein-DNA complex, the electrophoresis gel contents were transferred to a nitrocellulose membrane (GE Healthcare Life Sciences, UK) for western blotting. The membrane was blocked with 5% BSA (bovine serum albumin) for 1 h at room temperature. Then, rabbit monoclonal anti-RXRA (ab125001; Abcam) was added and incubated at 4°C overnight (dilution at 1:1,000). Peroxidase-conjugated anti-rabbit secondary antibody (1:5,000) was added the next day, and visualization was performed by enhanced chemiluminescence (Pierce).

### ChIP-qPCR Assay

HeLa cells were cross-linked with 1% formaldehyde for 10 min. Immunoprecipitation was performed using an EZ-Magna ChIP kit (Millipore, Massachusetts, USA). Chromatin fragments of 200–400 bp were incubated with 10 μl of anti-RXRA (Rb, ab125001; Abcam) at 4°C overnight. Five microliters of non-specific IgG and RNA Pol II (provided in the kit) were used as negative and positive controls, respectively. One-tenth of the volume of the supernatant containing the chromatin fragments was retained as input for normalization correction. Protein-enriched fragments were quantified by RT-PCR. The primer pairs used are shown in Supplementary Table 2.

### Statistical Analysis

Data were analyzed by SPSS (version 22.0, IBM, Armonk, NY) and GraphPad Prism (version 6.0, GraphPad, La Jolla, CA). The chi-square test was used to assess the differences in genotype distribution between normal controls and patients. The association between the HES1 promoter variant and CHD was evaluated by logistic regression analysis, and the results are shown as odds ratios (ORs) and 95% confidence intervals (CIs). Student's *t*-test was also used for statistical analysis, as described in the figure legends.

## Results

### Identification of HES1 Promoter Variants in CHD Families

**Figure 1:**
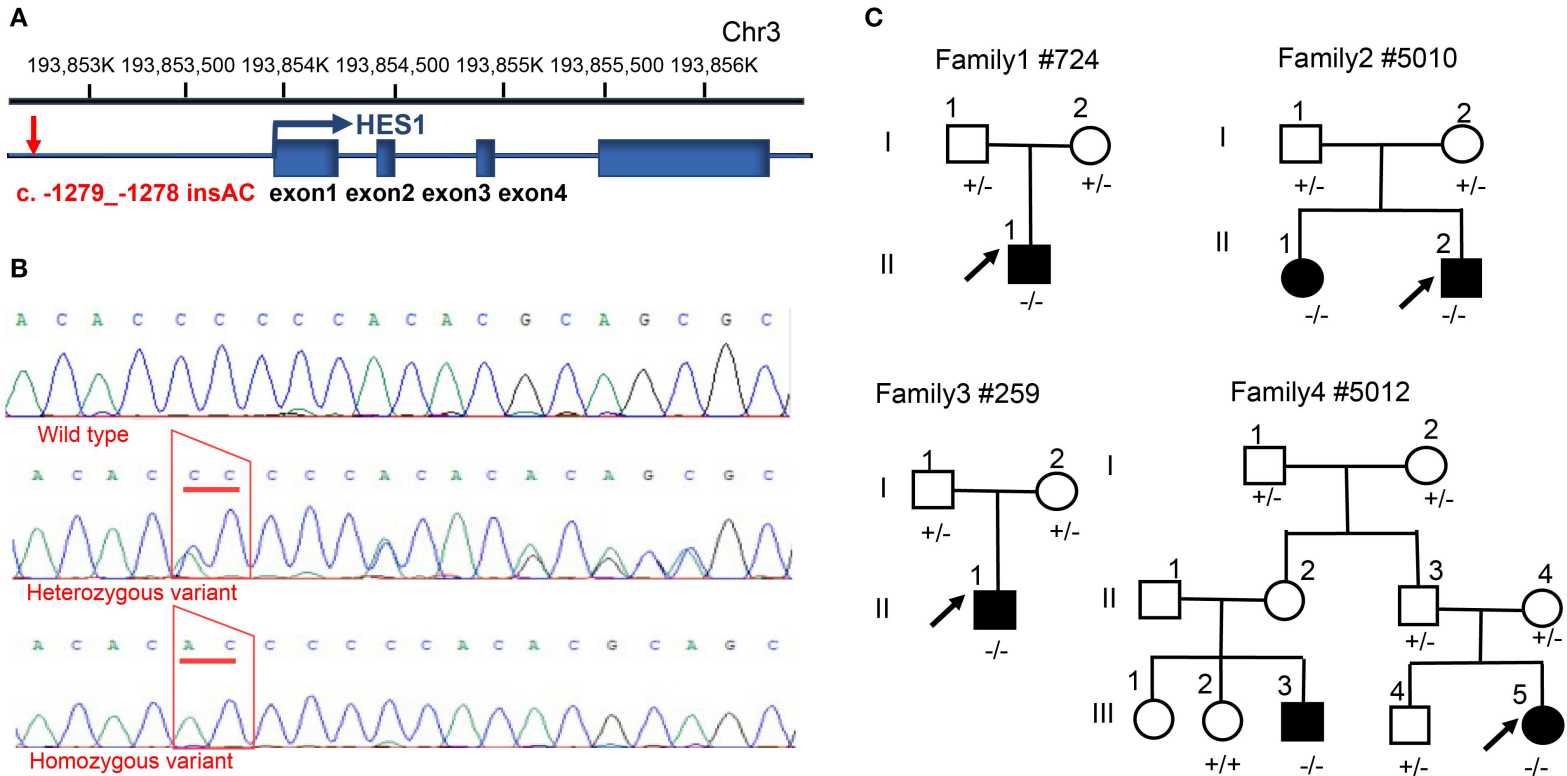
Identification of a HES1 variant in families with CHD. **(A)** Description of the location and structure of the HES1 gene on the chromosome and the relative position of the promoter variant. **(B)** Sanger sequencing of the homozygous and heterozygous sequence alterations in genomic DNA from a carrier and an unrelated control. The variant position is indicated by a red trapezoid. **(C)** Family pedigrees showing the phenotype and genotype of each family member; the arrow indicates the proband. Circles indicate female family members, and squares indicate male family members. - denotes the c. -1279_-1278 insAC variant; +/− indicates individuals carrying a heterozygous variant; and −/− indicates individuals with a homozygous variant.

To identify potential non-coding variants in the HES1 gene that could be responsible for CHD, we carried out direct Sanger sequencing of the promoter region of the HES1 gene in 12 unrelated families whose patients have been proven to carry no pathogenic coding variants in CHD-related genes (data not provided). The clinical phenotypes of the patients in these families are shown in Supplementary Table 4. Interestingly, a potential non-coding risk variant (NM_005524.4:c.-1279_-1278 insAC, rs148941464) was identified in the HES1 promoter region. This variant was located 1279 bp upstream of the transcription initiation site of the HES1 gene (Figures 1A,B). The results showed that 15 out of 16 (93.75%) CHD patients harbored homozygous variants in the HES1 promoter; no variant was observed in the remaining patient (family 6: II-1). Four out of the 12 CHD families (33.33%) showed cosegregation of homozygous variants and the disease phenotype (Figure 1C), indicating that the HES1 homozygous variant was overrepresented among the CHD patients. However, in other family members with a normal phenotype, we observed six individuals harboring the homozygous variant in five families (family 5:II-1, II-3; family 9:I-2; family 10:I-2; family 11:I-2; and family 12:I-2) (Supplementary Figures 1–3), suggesting incomplete penetrance; this observation is consistent with previous findings of incomplete penetrance observed in the analysis of WES data in CHD (Page et al., 2019). Moreover, the HES1 gene was predicted to be likely to exhibit haploinsufficiency in consented DECIPHER data (%HI: 11.44); thus, we concluded that the HES1 homozygous variant is a risk genetic factor underlying CHD.

### Association of the HES1 Promoter Variant With the Risk of CHD Development

To further investigate the relationship between the HES1 promoter variant and the risk of CHD development, we then recruited an additional 629 sporadic non-syndromic CHD cases and 696 healthy controls and performed direct Sanger sequencing of the HES1 promoter to identify heterozygous and homozygous variants. As expected, we observed a significant association between the homozygous HES1 promoter variant and disease phenotype (18.92% of cases vs. 9.91% of controls; OR: 2.291, 95% CI: 1.637–3.207, *p* = 9.72 × 10^−7^, Table 2) in the CHD cases and healthy controls. No significant association with CHD was obtained for the heterozygous variant of the HES1 promoter (*p* > 0.05) in this study. Considering the discordant phenotypes of different subtypes in the CHD cohort, we performed further association analysis tests of the HES1 homozygous variant with each subtype included in the CHD cohort in this study. Interestingly, the HES1 homozygous variant was strongly associated with transposition of the great arteries (TGA) (OR: 3.726, 95% CI: 1.637–3.207, *p* = 0.0003). Moreover, the prevalence of TGA patients with HES1 homozygous variants (27.66%) was also significantly higher than that for other subtypes or controls (Table 3). To further verify this result, we tested the HES1 variant in an additional 64 TGA cases and found that the homozygous variant was present at a similar proportion (23.44%) and was correlated with the occurrence of the disease (Supplementary Table 5). Taken together, our findings reveal that this homozygous variant in the HES1 promoter is significantly associated with an increased risk of CHD development, especially TGA.

**Table 2:** Association analyses of HES1 promoter variants with CHD in CHD cases and controls.

**Group (Phenotype)**	**Genetic model**	**Genotypes**	**Cases *N* (%)**	**Controls *N* (%)**	**OR (95% CI)**	***P*-value**	**HWE *P*-value**
**CHD (*****N*** **=** **629) vs. control (*****N*** **= 696)**							
	Co-dominant	Wt	268 (42.61)	356 (51.15)	1.00 (Ref)		
		Hete	242 (38.47)	271 (38.94)	1.186 (0.938–1.5)	0.154	
		Homo	119 (18.92)	69 (9.91)	2.291 (1.637–3.207)	9.72 × 10^−7^	
	Allele	Major allele	778 (61.84)	983 (70.62)	1.00 (Ref)		
		Minor allele	480 (38.16)	409 (29.38)	1.483 (1.261–1.744)	2.0 × 10^−6^	0.516

*Ref, reference; OR, odds ratio; CI, confidence interval; wt, wild type; hete, heterozygous variant; homo, homozygous variant*.

*OR and 95% CI calculations were conducted under the assumption that variant alleles were risk alleles*.

*The P-value for the chi-square test and HWE P-value for the Hardy-Weinberg equilibrium test in the control subjects*.

**Table 3:** Association analyses of HES1 promoter variants with CHD risk in various CHD subtypes.

**Group (Phenotype)**	**Genetic model**	**Genetype**	**Cases *N* (%)**	**Controls *N* (%)**	**OR (95% CI)**	***P*-value**
**TOF (*****N*** **= 96) vs. control (*****N*** **= 696)**						
	Co-dominant	Wt	41 (42.71)	356 (51.15)	1.00 (Ref)	
		Hete	41 (42.71)	271 (38.94)	1.314 (0.829–2.083)	0.245
		Homo	14 (14.58)	69 (9.91)	1.762 (0.911–3.406)	0.089
	Allele model	Major allele	123 (64.06)	983 (70.62)		
		Minor allele	69 (35.94)	409 (29.38)	1.134 (0.982–1.850)	0.064
**TGA (*****N*** **= 47) vs. control (*****N*** **= 696)**						
		Wt	18 (38.3)	356 (51.15)	1.00 (Ref)	
		Hete	16 (34.04)	271 (38.94)	1.168 (0.585–2.332)	0.66
		Homo	13 (27.66)	69 (9.91)	3.726 (1.745–7.956)	0.000322
	Allele model	Major allele	52 (55.32)	983 (70.62)		
		Minor allele	42 (44.68)	409 (29.38)	1.941 (1.272–2.962)	0.001794
**PA (*****N*** **= 37) vs. control (*****N*** **= 696)**						
	Co-dominant	Wt	18 (48.65)	356 (51.15)	1.00 (Ref)	
		Hete	13 (35.14)	271 (38.94)	0.949 (0.457–1.970)	0.888
		Homo	6 (16.22)	69 (9.91)	1.720 (0.659–4.488)	0.263
	Allele model	Major allele	49 (66.22)	983 (70.62)		
		Minor allele	25 (33.78)	409 (29.38)	1.226 (0.747–2.012)	0.419
**DORV (*****N*** **= 13) vs. control (*****N*** **= 696)**						
	Co-dominant	Wt	6 (46.15)	356 (51.15)	1.00 (Ref)	
		Hete	4 (30.77)	271 (38.94)	0.876 (0.245–3.134)	0.838
		Homo	3 (23.08)	69 (9.91)	2.580 (0.630–10.563)	0.172
	Allele model	Major allele	16 (61.54)	983 (70.62)		
		Minor allele	10 (38.46)	409 (29.38)	1.502 (0.676–3.338)	0.315
**other CTD (*****N*** **= 18) vs. control (*****N*** **= 696)**						
	Co-dominant	Wt	8 (44.44)	356 (51.15)	1.00 (Ref)	
		Hete	6 (33.33)	271 (38.94)	0.985 (0.338–2.873)	0.978
		Homo	4 (22.22)	69 (9.91)	2.580 (0.756–8.805)	0.117
	Allele model	Major allele	22 (61.61)	983 (70.62)		
		Minor allele	14 (38.38)	409 (29.38)	1.529 (0.775–3.019)	0.217
**VSD (*****N*** **= 293) vs. control (*****N*** **= 696)**						
	Co-dominant	Wt	126 (43.0)	356 (51.15)	1.00 (Ref)	
		Hete	111 (37.88)	271 (38.94)	1.157 (0.857–1.562)	0.34
		Homo	56 (19.11)	69 (9.91)	2.293 (1.527–3.444)	0.00005
	Allele model	Major allele	363 (61.94)	983 (70.62)		
		Minor allele	223 (38.05)	409 (29.38)	1.476 (1.206–1.808)	0.000159
**ASD (*****N*** **= 101) vs. control (*****N*** **= 696)**						
	Co-dominant	Wt	39 (38.61)	356 (51.15)	1.00 (Ref)	
		Hete	39 (38.61)	271 (38.94)	1.314 (0.820–2.104)	0.255
		Homo	23 (22.77)	69 (9.91)	3.043 (1.710–5.414)	0.000088
	Allele model	Major allele	117 (57.92)	983 (70.62)		
		Minor allele	85 (42.07)	409 (29.38)	1.746 (1.291–2.362)	0.000266
**VSD+ASD (*****N*** **= 31) vs. control (*****N*** **= 696)**						
	Co-dominant	Wt	12 (38.71)	356 (51.15)	1.00 (Ref)	
		Hete	12 (38.78)	271 (38.94)	1.314 (0.581–2.970)	0.511
		Homo	7 (22.59)	69 (9.91)	3.01 (1.144–7.917)	0.02
	Allele model	Major allele	36 (58.00)	983 (70.62)		
		Minor allele	26 (41.99)	409 (29.38)	1.736 (1.035–2.912)	0.035

### Pathogenic Evidence of the Homozygous HES1 Promoter Variant in CHD Patients

**Figure 2:**
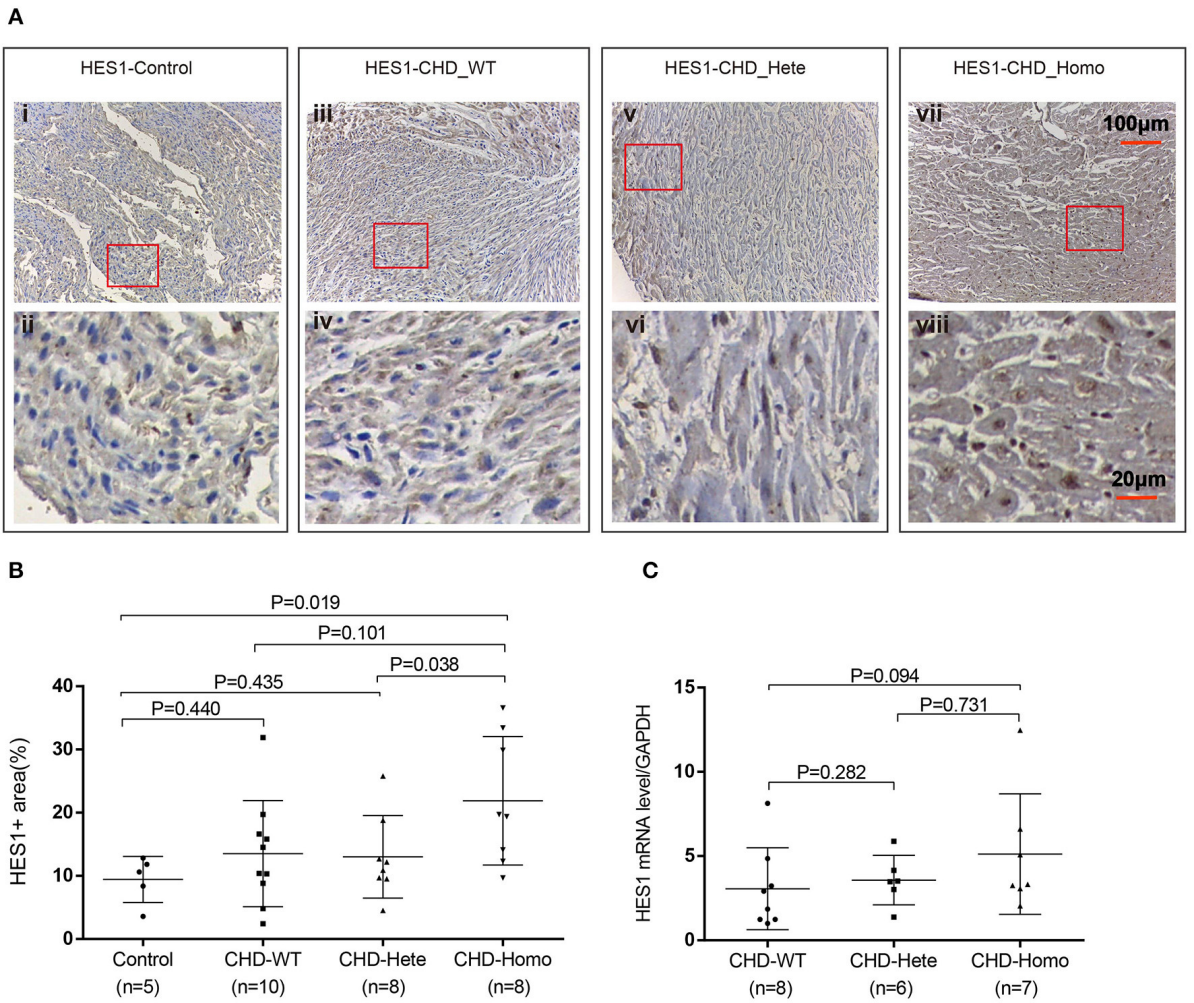
HES1 expression is significantly elevated in patients with homozygous variants. **(A,B)** Patients with the homozygous variant showed significantly increased HES1 protein levels in right ventricular outflow tract tissues compared to normal controls (*P* = 0.019). Patients with the heterozygous variant did not show a significant increase. **(C)** RT-PCR was used to detect the expression of HES1 mRNA. The results showed a non-significant increase in the expression level of HES1 in the patients with the homozygous variant compared with that of the patients without the variant (*P* = 0.094). The changes in protein and mRNA levels were consistent. *P*-values for the Mann-Whitney U test; *P*-values <0.05 were considered significant.

To investigate the effect of the HES1 promoter variant on gene expression, we carried out IHC to evaluate the immune reactivity of an antibody against HES1 in human right ventricular outflow tract tissues (RVOTs) obtained from CHD patients and normal controls (most of the tissues that could be obtained during surgery came from TOF patients). The phenotype and genotype of the CHD patients and IHC controls are shown in Supplementary Tables 6, 7. The immune reactivity in the RVOT of patients carrying the homozygous HES1 promoter variant was significantly higher than that in normal controls (*P* = 0.019) (Figures 2A,B). At this level of analysis, we found a trend of increased HES1 protein levels in patients with the homozygous variant compared to the patients harboring a heterozygous variant. A similar trend of increased HES1 levels in patients with the heterozygous variant compared to patients without the variant was observed; however, neither difference reached significance (*P* = 0.038, *P* = 0.101). In addition, RT-PCR analysis of the HES1 gene in the RVOT of CHD patients indicated a trend of increased HES1 mRNA expression in the patients with the homozygous variant compared with that of patients without the variant, though again the trend was not statistically significant (Figure 2C). These findings suggested that the homozygous HES1 promoter variant is responsible for increased expression of HES1 in CHD patients.

### Effect of Homozygous HES1 Promoter Variant on Gene Activity

**Figure 3:**
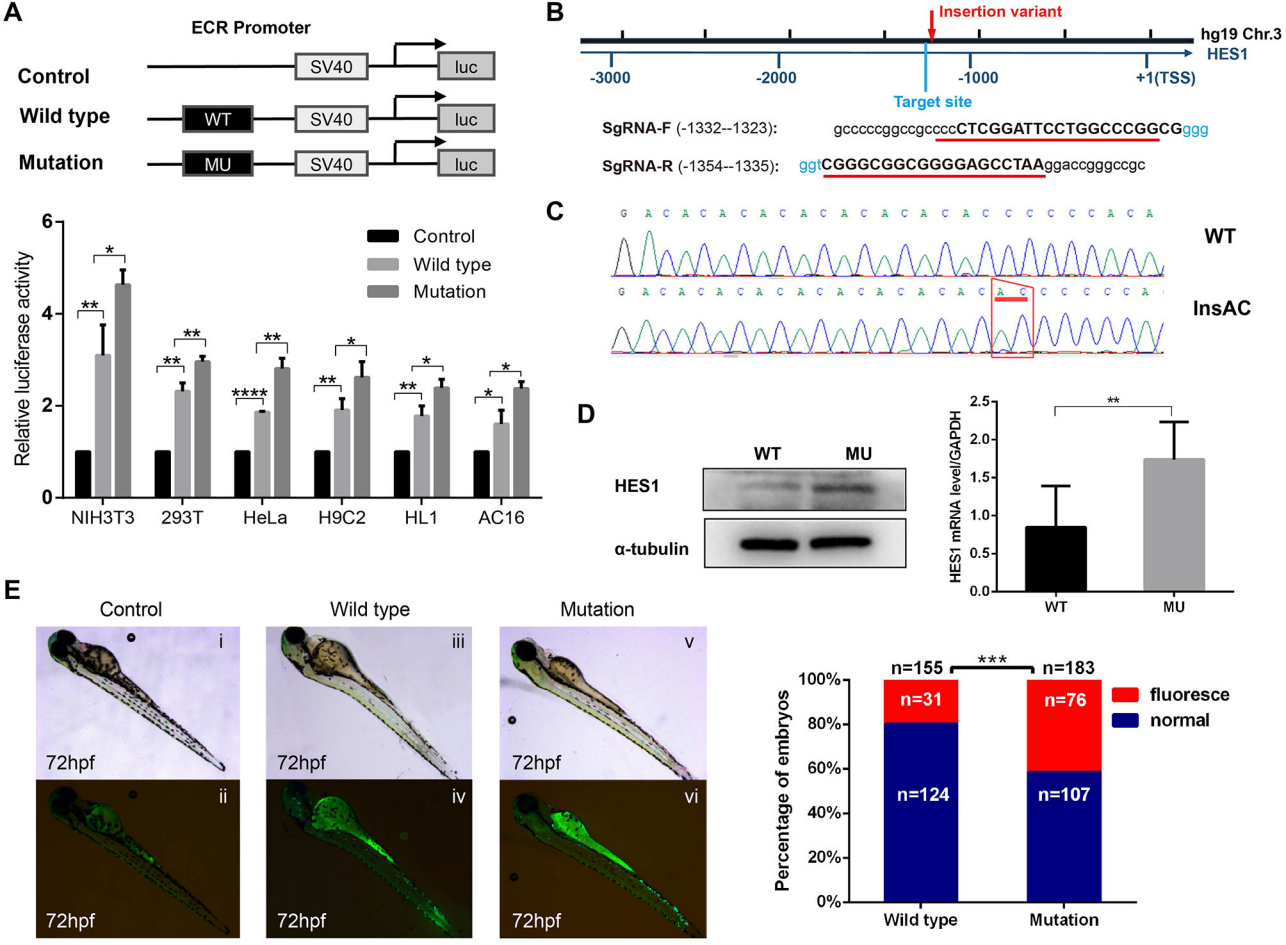
The HES1 promoter variant increases gene activity. **(A)** Constructs and vector plasmids with wild-type and mutant ECR fragments were transfected into six cell lines and assayed for luciferase reporter gene activity. The results showed that the luciferase activity of the construct with the mutant ECR was significantly increased compared with that of the wild-type. Data are expressed as the mean ± SD of three independent experiments. *p < 0.05, **p < 0.01, and ****P < 0.0001 by Student's *t*-test. **(B)** Schematic diagram of the position of the HES1 promoter variation and the SgRNA sequence for the target site. **(C)** Sanger sequencing confirmed the insertion mutation (c.−1279−1278 insAC). **(D)** Western blot and Q-PCR results showed that the expression of HES1 significantly increased in HES1 promoter variant HeLa cells. Data are expressed as the mean ± SD of three independent experiments. **p < 0.01 by Student's *t*-test. **(E)** A regulatory variant in the HES1 ECR increased the activity of the enhancer. pCNE-HES1-WT or pCNE-HES1-MU was injected into zebrafish with Tol2 mRNA. The enhancer activity was further increased by the variant mutation. (i,ii) Control zebrafish without vector injection. (iii,iv) Wild-type zebrafish injected with pCNE-HES1-WT. (v,vi) Mutant zebrafish injected with pCNE-HES1-MU. The statistical analysis of the percentage of zebrafish exhibiting enhancer activity after microinjection showed that a significantly higher proportion of the zebrafish injected with the mutant vector exhibited enhancer activity than the wild-type zebrafish. ***P < 0.001 by chi-square test.

To further explore the influence of the homozygous HES1 promoter variant, we performed luciferase assays to examine the effect of the promoter variant on gene activity in six different cell lines (NIH3T3, 293T, HeLa, H9C2, HL1, and AC16). As shown in Figure 3A, the expression of the reporter gene was significantly higher in the cells transfected with the recombinant plasmid harboring the HES1 promoter variant than in the wild-type cells, indicating that the HES1 promoter variant can increase the gene activity (*P* < 0.05 for NIH3T3, H9C2, HL1, and AC16; *P* < 0.01 for 293T, and HeLa). In addition, we used CRISPR–Cas9 technology to generate a HES1 promoter variant knock-in HeLa cell line and found that the expression of HES1 was significantly increased in this cell line (Figures 3B–D).

Additionally, we used a zebrafish experimental model to assess the influence of this HES1 promoter variant on gene activity *in vivo* and found that the level of luminescence in the promoter variant zebrafish was significantly higher than that in the controls (*P* < 0.001) (Figure 3E), suggesting that this HES1 promoter variant was able to enhance gene expression *in vivo*.

### Regulatory Mechanism of Homozygous HES1 Promoter Variant

**Figure 4:**
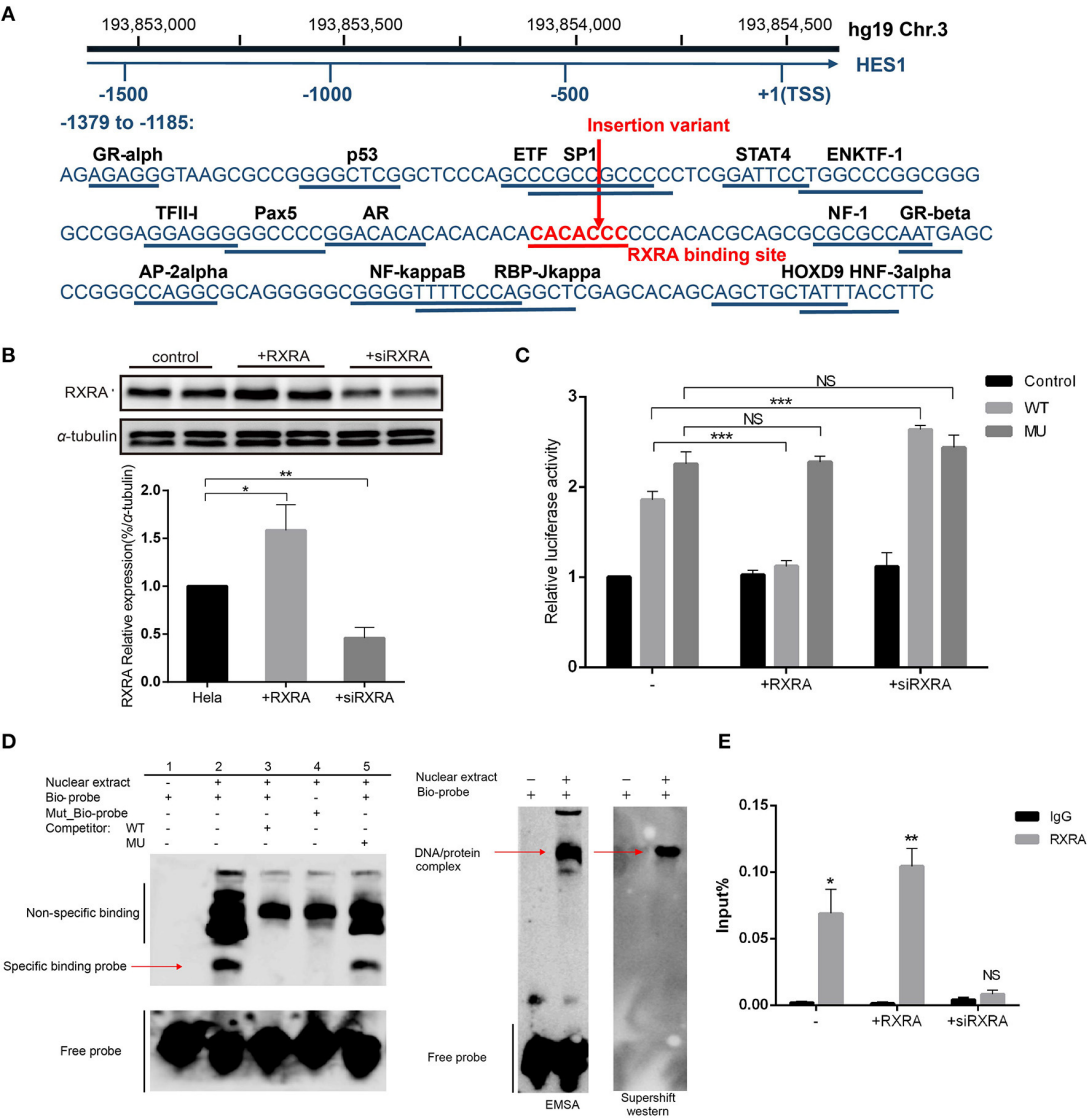
The variant affects the inhibitory effect of RXRA on enhancer activity. **(A)** The variant nucleotide (boxed) is highly conserved and overlaps a predicted conserved binding site for RXRA. **(B)** Western blot results confirmed the successful overexpression or inhibition of the expression of RXRA in HeLa cells. Data are expressed as the mean ± SD of three independent experiments. *p < 0.05 and **p < 0.01 by Student's *t*-test. **(C)** In HeLa cells, luciferase constructs with wild-type and mutant fragments were cotransfected into cells with a human RXRA expression vector and RXRA siRNA, and the corresponding luciferase activity was analyzed. The variant affected the binding of the transcription factor RXRA to the enhancer so that the inhibitory effect was relieved, and the activity of the enhancer increased. Cotransfection with RXRA siRNA partially abolished the inhibition of enhancer activity. Data are expressed as the mean ± SD of three independent experiments. ***P < 0.001 by Student's *t*-test. **(D)** An electrophoretic mobility shift assay (EMSA) showed high-affinity, sequence-specific interaction of HES1 with a double-stranded oligonucleotide containing the wild-type (wt) sequence but not the mutant sequence (mu). The shifted signal was suppressed by the addition of an unlabeled consensus high-affinity binding site for HES1. The red arrow indicates the shifted HES1 complex. The areas indicated by the black lines represent non-specific probe binding and free probe. EMSA and supershift western blot analyses confirmed RXRA protein binding. The arrows indicate the complex containing the biotin-labeled HES1 probe and RXRA protein. **(E)** ChIP-qPCR confirmed that RXRA was enriched in the HES1 promoter (near the HES1 promoter variant).

We further explored the mechanism by which the HES1 promoter variant regulates gene expression. Using the PROMO database (http://alggen.lsi.upc.es/), we screened the variant-containing DNA sequence region and identified a conserved potential binding site for RXRA (T01345) (Figure 4A) in this region.

We performed luciferase assays in cells with RXRA overexpression or RXRA siRNA and the wild-type or pGL3-promoter recombinant plasmids containing the variant. The results showed that increased RXRA protein was able to significantly inhibit gene activity (*P* < 0.0001), and knockdown of RXRA was able to increase gene activity (*P* < 0.0001) (Figure 4C). These results confirmed the role of RXRA in negatively regulating HES1 expression. Interestingly, the luciferase activity showed no significant difference between the cells transfected with pGL3-promoter recombinant plasmids containing the HES1 variant and the wild-type cells in the presence or absence of RXRA protein (Figure 4C). Consistently, similar results were observed in other cell lines (Supplementary Figure 5), indicating that the HES1 variant abrogated the effects of RXRA on HES1 gene activity by disturbing the binding of RXRA to the enhancer.

To further confirm whether the variant disrupted the binding of RXRA to the enhancer, we performed EMSAs using the RXRA protein and a biotin-labeled probe (HES1: −1317 to −1278) or a mutated biotin-labeled probe. As shown in Figure 4D, the biotin-labeled probe bound the RXRA protein *in vitro* (Figure 4D, lane 2). However, this band was diminished when a wild-type competitor was added or a mutated biotin-labeled probe was used, indicating that the binding was disrupted (Figure 4D, lane 3, lane 4). The binding of RXRA to the biotin-labeled probe was not changed when the mutant competitor probe was added (Figure 4D, lane 5). These results confirmed that RXRA can bind to the enhancer region and that the identified HES1 promoter variant disrupted the binding of RXRA. A supershift western blot was performed to verify that the shift was caused by RXRA (Figure 4D). In addition, we also directly confirmed that RXRA is enriched near the HES1 promoter variant site by ChIP-qPCR (Figure 4E).

## Discussion

CHD occurs as a result of abnormalities in cardiac development during embryogenesis. These abnormalities are known to be highly related to changes in cardiogenic transcription factors and other developmental pathways that coordinate the development of the heart, as indicated by the increasing number of CHDs identified to be associated with mutations or genetic variants in coding regions of cardiac development-related genes (Schott et al., 1998; Garg et al., 2003; Bruneau, 2008; Lopes et al., 2011; Zaidi and Brueckner, 2017). However, these examples can explain only a small number of CHD cases. The currently accepted hypothesis is that CHD is caused by the interactions between genetic variants and multiple susceptibility factors.

Simultaneous assessment of an entire patient exome and identification of causal genetic variations has become possible thanks to advances in DNA sequencing technology. However, a main limitation of this approach is that the mutation investigation area is limited to only the 1–2% of the whole genome that encodes proteins (Bamshad et al., 2011). The influence of genetic variation in non-coding sequences on the etiology of complex diseases has been recognized. Genome-wide association studies (GWASs) have shown that a large number of non-coding variants account for increased risks of various common diseases, usually by destroying cis-regulatory elements (CREs) that influence the expression levels of nearby genes (Visel et al., 2009; Musunuru et al., 2010; Harismendy et al., 2011; Sakabe et al., 2012). Non-coding variants might account for some congenital malformations, including CHD; however, to the best of our knowledge, this has not been extensively investigated. Mutations truncating the mRNA or modifying the structure or amino acid composition of a transcription factor could cause severe morphological phenotypes, similar to those of CHD. However, it is not clear whether mutations in the CREs of these developmental genes can lead to deleterious effects. Previous studies have found variations in CREs that affect CHD development. Non-coding mutations in TBX5 cardiac enhancers were found to cause a large number of CHDs associated with TBX5 dysfunction, effectively decoupling the heart, and hand phenotype of Holt-Oram syndrome (Smemo et al., 2012). Another study found that patients from two families with CHD carried a very similar ~1 Mb deletion upstream of SOX9; the destruction of cardiac enhancers upstream of SOX9 may be responsible for human CHD (Sanchez-Castro et al., 2013).

In our study, we found a mutation in the *HES1* promoter in a family with CHD. Almost all probands (93.75%) carry this mutation, suggesting that this variant may be related to the occurrence of CHD. In a subgroup analysis, we found that the homozygous variant was significantly associated with increased CHD. Raetzman et al. noted that HES1 plays a central role in the proliferation and differentiation of a series of cell types and that it is essential for maintaining progenitor cells in an undifferentiated state (Raetzman et al., 2007). Rochais et al. found that *Hes1* mutant embryos at day 15.5 had outflow tract alignment defects, including ventricular septal defects and overriding aortas (Rochais et al., 2009). At earlier developmental stages, SHF proliferation and the number of cardiac neural crest cells were repressed, and the outflow tract could not be completely extended, which indicates that HES1 is necessary for the development of the cardiac arteries. A study by van Bueren et al. found that Hes1 mutant mice exhibited a range of partially penetrant 22q11DS-like defects, including pharyngeal arch artery (PAA), outflow tract, craniofacial and thymic abnormalities (van Bueren et al., 2010). These findings suggest that HES1 is closely related to the development of the cardiac outflow tract. CHD is a structural abnormality caused by the malformation or abnormal development of the heart and large blood vessels during embryonic development, so it is reasonable that HES1 is closely related to the occurrence of CHD.

Many studies have reported that abnormal expression of HES1 is also closely related to the occurrence of CHD. One study demonstrated that overexpression of HES1 can increase apoptosis and inhibit cell proliferation and that miR-182 exerted a protective effect by suppressing HES1 in cardiomyocytes exposed to hypoxia (Zhang et al., 2018). Wu K et al. found that HES1 expression was elevated in CHD model mice and that the activation of the NOTCH signaling pathway may lead to CHD (Wu et al., 2018).

In our study, we further examined the protein and mRNA levels of HES1 in RVOT from CHD patients with different genotypes and normal controls. Then, we found that the expression of HES1 in patients with homozygous mutations was significantly higher than that in wild-type CHD patients and normal controls. The results of others' research are consistent with our observations. Because this variant is located in the *HES1* promoter, we speculated that it may increase the expression of HES1 by affecting the transcription of this gene, thereby activating the NOTCH signaling pathway to disturb the normal growth and development of the cardiac outflow tract.

To test this hypothesis, we performed a series of experiments in cells and showed that the activity of the mutant enhancer was significantly higher than that of the wild-type enhancer. The effect of this *HES1* promoter variant on the activity of the fragment was directly confirmed. In addition, to observe whether the HES1 promoter variant directly affects the expression of endogenous HES1, we used the CRISPR–Cas9 system to construct a mutant cell line of variant knock-in HeLa cells. The results showed that the mRNA and protein levels of HES1 were significantly higher in the mutant cell line than in the wild-type cell line. This shows that the HES1 promoter variant can indeed upregulate the expression of endogenous HES1.

We also conducted luciferase experiments with recombinant plasmids containing wild-type and mutant HES1 promoter fragments. We found that the wild-type fragment has a transcription-enhancing activity and that the variant further increased the transcriptional activity from the recombinant plasmids. Therefore, we confirmed the effect of the variant on the transcriptional activity of the promoter *in vitro* at the cellular level. To further explore the effect of the variant on the transcriptional activity of the promoter in animals, we constructed recombinant plasmids to drive GFP expression under the control of the wild-type and mutant HES1 promoter fragments and injected this construct into zebrafish embryos. Then, we observed the expression site and expression intensity (luminescence ratio) of GFP driven by the recombinant plasmids in zebrafish, specifically, the enhancer effect of this fragment, whether it can drive the expression of GFP in the heart, and whether the variant influenced this effect. We observed GFP expression in the heart after 72 h, at which stage the zebrafish heart has developed and has begun to differentiate and proliferate (Stainier, 2001; Ackermann and Paw, 2003). In some zebrafish embryos, GFP was also expressed in the forebrain, notochord, and blood. However, constructs with mutant fragments increased the proportion of zebrafish expressing GFP in the heart, suggesting that this *HES1* promoter variant can enhance cardiac-specific expression of this fragment.

Retinoid X receptors (RXRs) are nuclear receptors that act as transcription factors by binding to specific sequences in target gene promoters, thereby participating in retinoid-mediated gene activation to mediate the biological effects of retinoids (Evans and Mangelsdorf, 2014; Piskunov et al., 2014). The retinoic acid (RA) signaling pathway has been shown to play an important role in many aspects of cardiac development, including outflow tract development, suggesting an important role for RXRA in cardiac development (Cresci et al., 1999; Merki et al., 2005; Xavier-Neto et al., 2015; Stefanovic and Zaffran, 2017). Shantae et al. observed epicardial growth retardation in RXRA mutant embryos, leading to epicardial abnormalities and ultimately to cardiac malformations. According to *in silico* predictions, the transcription factor RXRA can bind to this site (Jenkins et al., 2005; Mascrez et al., 2009). Thus, *HES1* may be a downstream target gene of RXRA. Given the important role of the RA signaling pathway in cardiac development, we hypothesized that the susceptibility effect of the *HES1* promoter variant identified here can be explained by weakened RXRA regulation. Our luciferase and EMSA results indicate that this *HES1* promoter variant can alter the interaction between the RXRA transcription factor and sequence elements in the *HES1* promoter, eliminating the inhibitory effect of RXRA on enhancer activity. In addition, we also directly confirmed that RXRA is enriched near the HES1 promoter variant site by ChIP-qPCR. These results all support our hypothesis. Studies have reported that RXRA directly inhibits the expression of Fgf8 by recruiting the inhibitory histone marker H3K27me3 and polycomb inhibitor complex 2 (PRC2) and that the recruitment of these factors is RXRA dependent (Kumar and Duester, 2014). Therefore, we hypothesized that this mechanism was responsible for RXRA-mediated inhibition of *HES1* enhancer activity.

Our study found that the frequency of the major *HES1* enhancer allele in the normal population is 70.62%, while the frequency of the minor allele is 29.38%. Therefore, according to the law of genetic balance, the theoretical frequency of homozygosity produced by heterozygous carriers of this SNP in the population should be 8.63%. The actual frequency of homozygosity in the population is 9.91%. The Hardy-Weinberg balance test was performed, and *P* = 0.516, indicating that the control population is in equilibrium.

Our study is the first to show that RXRA inhibits the *HES1* enhancer and that a functional genetic variation in the *HES1* enhancer is associated with CHD. However, this study has certain limitations. First, the sample size of the population was relatively small, especially the number of families. In the future, the sample size needs to be expanded to further confirm the mutation risk. Moreover, the number of tissue samples was also small, and it is not yet possible to unambiguously confirm that the expression of HES1 is significantly increased in the heart tissues of patients with homozygous variants. Therefore, it is also necessary to expand the sample to confirm this conclusion. In addition, although our study found that a *HES1* promoter variant is associated with increased CHD, it is difficult to use animal model-based research to support direct correlation between the variant and the abnormal cardiac phenotype, largely due to the concern of the differences in genomics and the fine-tuning developmental mechanisms among different species. Thus, the pathogenic mechanism will be further explored in iPSC-based analysis in the future.

## Conclusion

In summary, our findings reveal that a non-coding homozygous variant in the HES1 promoter has gain-of-function effects and is associated with an increased risk of CHD development, especially the severe TGA subtype.

## Data Availability Statement

The original contributions presented in the study are included in the article/Supplementary Material, further inquiries can be directed to the corresponding authors.

## Ethics Statement

The studies involving human participants were reviewed and approved by Fudan University Children's Hospital Ethics Committee. Written informed consent to participate in this study was provided by the participants' legal guardian/next of kin. The animal study was reviewed and approved by Department of Laboratory Animal Science of the Fudan University.

## Author Contributions

GH and WS conceived the study and supervised the data analysis and manuscript completion. FW and MQ provided critical review and edited the manuscript. YS, WC, and ZH drafted the manuscript and analyzed the data. GT, ML, ZZ, ZF, FW, MQ, and XM collected the samples and clinical information. All authors contributed to and discussed the results and approved the final draft.

## Conflict of Interest

The authors declare that the research was conducted in the absence of any commercial or financial relationships that could be construed as a potential conflict of interest.
