# Supplementary material for: A Non-coding HES1 Variant Predisposes Children to Congenital Heart Disease in Chinese Population
Source: Front Cell Dev Biol. 2021 Jan 28;9:631942. doi: 10.3389/fcell.2021.631942 (PMC7876461; doi:10.3389/fcell.2021.631942)
Supplement: Supplementary file 1 [file Table_1.DOCX]

Supplementary Material

**Supplementary Table 1** **Characteristic of the CHD patients and controls.**

|  | **Control**  **N=5** | **Wild type**  **N=10** | **Heterozygous**  **N=8** | **Homozygous**  **N=8** |
| --- | --- | --- | --- | --- |
| **Sample for IHC** |  |  |  |  |
| Age, years (mean±SD) | 0.35 (±0.43) | 2.5 (±3.34) | 1.12 (±1.00) | 2.90 (±4.86) |
| Gender |  |  |  |  |
| Male, n (%) | 5 (100) | 3 (30.0) | 4 (50.0) | 5 (62.5) |
| Female, n (%) | 0 | 7 (70.0) | 4 (50.0) | 3 (37.5) |
| **Sample for RT-PCR** |  |  |  |  |
| Age, years (mean±SD) |  | 3.04 (±4.68) | 0.97 (±1.12) | 1.31 (±1.63) |
| Gender |  |  |  |  |
| Male, n (%) |  | 4 (50.0) | 3 (50.0) | 6 (85.7) |
| Female, n (%) |  | 4 (50.0) | 3 (50.0) | 1 (14.3) |

**Supplementary Table 2** **Primers used for qPCR, CRISPR/Cas9 construction, and genotyping in sample.**

| **Primer Name** | **Forward (5′→3′)** | **Reverse (5′→3′)** |
| --- | --- | --- |
| HES1-detect | GAAAACCCCAAGCCCGAAAG | ACCCCGTCTTTCAGAAATTCC |
| HES1-seq | CCCGCCGGGAAGAGCTGACC | GGTGGCGGGCAAGGAAGCAG |
| HES1-mRNA | AAAAATTCCTCGTCCCCGGT | ATGCCGCGAGCTATCTTTCT |
| RXRA-mRNA | TTAGGGACTGAAGCCCAGCA | GTGGAGAAATCTGCCCTGACT |
| GAPDH-mRNA | GGAGCGAGATCCCTCCAAAAT | GGCTGTTGTCATACTTCTCATGG |
| HES1-ECR | GGGGTACCGAAAACCCCAAGCCCGAAAG | CTAGCTAGCACCCCGTCTTTCAGAAATTCC |
| SgRNA-F | CACCG-CTCGGATTCCTGGCCCGGCG | AAAC-CGCCGGGCCAGGAATCCGAG-C |
| SgRNA-R | CACCG-AATCCGAGGGGGCGGCGGGC | AAAC-GCCCGCCGCCCCCTCGGATT-C |
| HES1-ECRm | ACACACACACACACACACACCCCCCAC | CCGGGGCCCCCTCCTCCG |
| Injection-Vector | AAGACGGGGTGATCTCTCGACTCTAGAGGGTATATAATGGATC | TGGGGTTTTCGGGCCCATCTGGCCTGTG |
| Injection-ECR | AGATGGGCCCGAAAACCCCAAGCCCGAAAG | TCGAGAGATCACCCCGTCTTTCAGAAATTC |
| Chip-RXRA-HES1 | GCCCCCTCGGATTCCT | CTCGAGCCTGGGAAAACC |

**Supplementary Table 3 Primer pairs for EMSA.**

| **Primer Name** | **Sequence (5′→3′)** |
| --- | --- |
| Biotin-HES1-RXRA-F | GCCGGAGGAGGGGGCCCCGGACACACACACACACACACCC |
| Biotin-HES1-RXRA-R | GGGTGTGTGTGTGTGTGTGTCCGGGGCCCCCTCCTCCGGC |
| Biotin-mutHES1-RXRA-F | GCCGGAGGAGGGGGCCCCGGACACACACACACACACACACCC |
| Biotin-mutHES1-RXRA-R | GGGTGTGTGTGTGTGTGTGTGTCCGGGGCCCCCTCCTCCGGC |
| Unlabeled-HES1-RXRA-F | GCCGGAGGAGGGGGCCCCGGACACACACACACACACACCC |
| Unlabeled-HES1-RXRA-R | GGGTGTGTGTGTGTGTGTGTCCGGGGCCCCCTCCTCCGGC |
| Unlabeled-mutHES1-RXRA-F | GCCGGAGGAGGGGGCCCCGGACACACACACACACACACACCC |
| Unlabeled-mutHES1-RXRA-R | GGGTGTGTGTGTGTGTGTGTGTCCGGGGCCCCCTCCTCCGGC |

**Supplementary Table 4 Characteristic of the CHD patients and controls.**

| **Family ID** | **Proband ID** | **Gender** | **Age (years)** | **Phenotype** | **Genotype** |
| --- | --- | --- | --- | --- | --- |
| 1 | II-1 | M | 0.21 | TOF | Homo |
| 2 | II-1 | F | 9.07 | VSD | Homo |
| 2 | II-2 | M | 3.27 | TOF | Homo |
| 3 | II-1 | M | 0.5 | TOF | Homo |
| 4 | III-3 | F | 2.7 | DORV, ASD, CAVC, Isolated Dextrocardia | Homo |
| 4 | III-5 | M | 1.37 | VSD, ASD, PDA | Homo |
| 5 | III-2 | F | 4.59 | TOF | Homo |
| 5 | III-3 | M | 3.84 | VSD | Homo |
| 6 | II-1 | M | 9.45 | PS | WT |
| 6 | II-2 | M | 3.05 | PS | Homo |
| 7 | II-1 | M | 1.49 | DORV, TGA, VSD | Homo |
| 8 | II-1 | M | 16.6 | DORV, TGA, ASD, VSD | Homo |
| 9 | II-1 | M | 0.003 | DORV, TGA, ASD, VSD | Homo |
| 10 | II-1 | M | 0.21 | DORV, VSD, ASD | Homo |
| 11 | II-1 | M | 0.52 | TOF, PFO | Homo |
| 12 | II-1 | F | 0.21 | PA, VSD, PDA, PFO | Homo |

Abbreviations: TOF, Tetralogy of Fallot; VSD, Ventricular septal defect; DORV, Right double outlet right ventricle; ASD, Atrial septal defect; CAVC, common atrioventricular canal; PDA, patent ductus arteriosus; PS, pulmonary stenosis; TGA, transposition of the great arteries; PFO, patent foramen ovale; PA, pulmonary atresia.

**Supplementary Table 5** **Association analyses between addition HES1 promoter variant and CHD risk in TGA case and controls.**

| **Group (Phenotype)** | **Genetic model** | **Genotypes** | **Cases N (%)** | **Controls N (%)** | **OR (95% CI)** | **P-value** |
| --- | --- | --- | --- | --- | --- | --- |
| **TGA (N=64) vs control (N=696)** | | | | | | |
|  | Co-dominant | wt | 24 (37.5) | 356 (51.15) | 1.00 (Ref) |  |
|  |  | hete | 25 (39.06) | 271 (38.94) | 1.368 (0.765-2.449) | 0.289 |
|  |  | homo | 15 (23.44) | 69 (9.91) | 3.225 (1.61-6.459) | 5.61×10^-4^ |
|  | Allele | major allele | 73 (57.03) | 983 (70.62) | 1.00 (Ref) |  |
|  |  | minor allele | 55 (42.97) | 409 (29.38) | 1.811 (1.253-2.618) | 0.001402 |

**Supplementary Table 6** **Phenotype and genotype of the CHD patients for IHC.**

| **Group** | **Proband ID** | **Gender** | **Age (years)** | **Phenotype** | **Genotype** |
| --- | --- | --- | --- | --- | --- |
| CHD-WT | 6 | F | 7.34 | VSD | WT |
|  | 7 | M | 1.17 | VSD, RVAB, PS, LSVC, MS | WT |
|  | 8 | F | 0.79 | TOF, ASD, LSVC | WT |
|  | 9 | F | 0.09 | TOF | WT |
|  | 10 | M | 0.58 | VSD, DCRV | WT |
|  | 11 | F | 9.66 | D-TGA, VSD, PS | WT |
|  | 12 | F | 0.65 | TOF, LSVC, PFO | WT |
|  | 13 | M | 0.61 | TOF, PFO | WT |
|  | 14 | F | 0.6 | TOF, ASD, LSVC | WT |
|  | 15 | F | 3.46 | TOF | WT |
| CHD-Hete | 16 | F | 0.24 | TOF, ASD | Hete |
|  | 17 | M | 2.10 | TOF | Hete |
|  | 18 | F | 0.59 | TOF, PFO | Hete |
|  | 19 | M | 3.08 | TOF, PFO | Hete |
|  | 20 | M | 0.60 | TOF | Hete |
|  | 21 | F | 0.52 | TOF, PFO | Hete |
|  | 22 | F | 0.47 | TOF | Hete |
|  | 23 | M | 1.35 | TOF | Hete |
| CHD-Homo | 24 | F | 0.67 | TOF, DA | Homo |
|  | 25 | F | 0.22 | PA, VSD, PDA, XPFO | Homo |
|  | 26 | M | 14.29 | VSD, LSVC | Homo |
|  | 27 | M | 0.52 | TOF, ASD | Homo |
|  | 28 | M | 0.41 | CAVC | Homo |
|  | 29 | M | 4.93 | DORV, VSD, ASD | Homo |
|  | 30 | M | 0.52 | TOF, PFO | Homo |
|  | 31 | F | 1.66 | TOF | Homo |

**Supplementary Table 7** **Phenotype and genotype of the CHD patients for RT-PCR.**

| **Group** | **Proband ID** | **Gender** | **Age (years)** | **Phenotype** | **Genotype** |
| --- | --- | --- | --- | --- | --- |
| CHD-WT | 1 | F | 0.79 | TOF, ASD, LSVC | WT |
|  | 2 | M | 13.92 | TOF | WT |
|  | 3 | M | 0.36 | TOF, PS | WT |
|  | 4 | F | 0.65 | TOF, LSVC, PFO | WT |
|  | 5 | M | 0.67 | TOF, PFO | WT |
|  | 6 | F | 0.6 | TOF, ASD, LSVC | WT |
|  | 7 | F | 3.46 | TOF | WT |
|  | 8 | M | 0.47 | TOF | WT |
| CHD-Hete | 9 | M | 0.33 | PA, VSD, ASD | Hete |
|  | 10 | F | 0.05 | TOF, PDA, PFO | Hete |
|  | 11 | M | 3.08 | TOF, PFO | Hete |
|  | 12 | F | 0.52 | TOF, PFO | Hete |
|  | 13 | F | 0.47 | TOF | Hete |
|  | 14 | M | 1.35 | TOF | Hete |
| CHD-Homo | 15 | F | 0.22 | PA, VSD, PDA, PFO | Homo |
|  | 16 | M | 0.52 | TOF, ASD | Homo |
|  | 17 | M | 0.96 | TOF | Homo |
|  | 18 | M | 4.93 | DORV, VSD, ASD | Homo |
|  | 19 | M | 0.52 | TOF, PFO | Homo |
|  | 20 | M | 0.79 | TOF, DA | Homo |
|  | 21 | M | 1.24 | TOF | Homo |


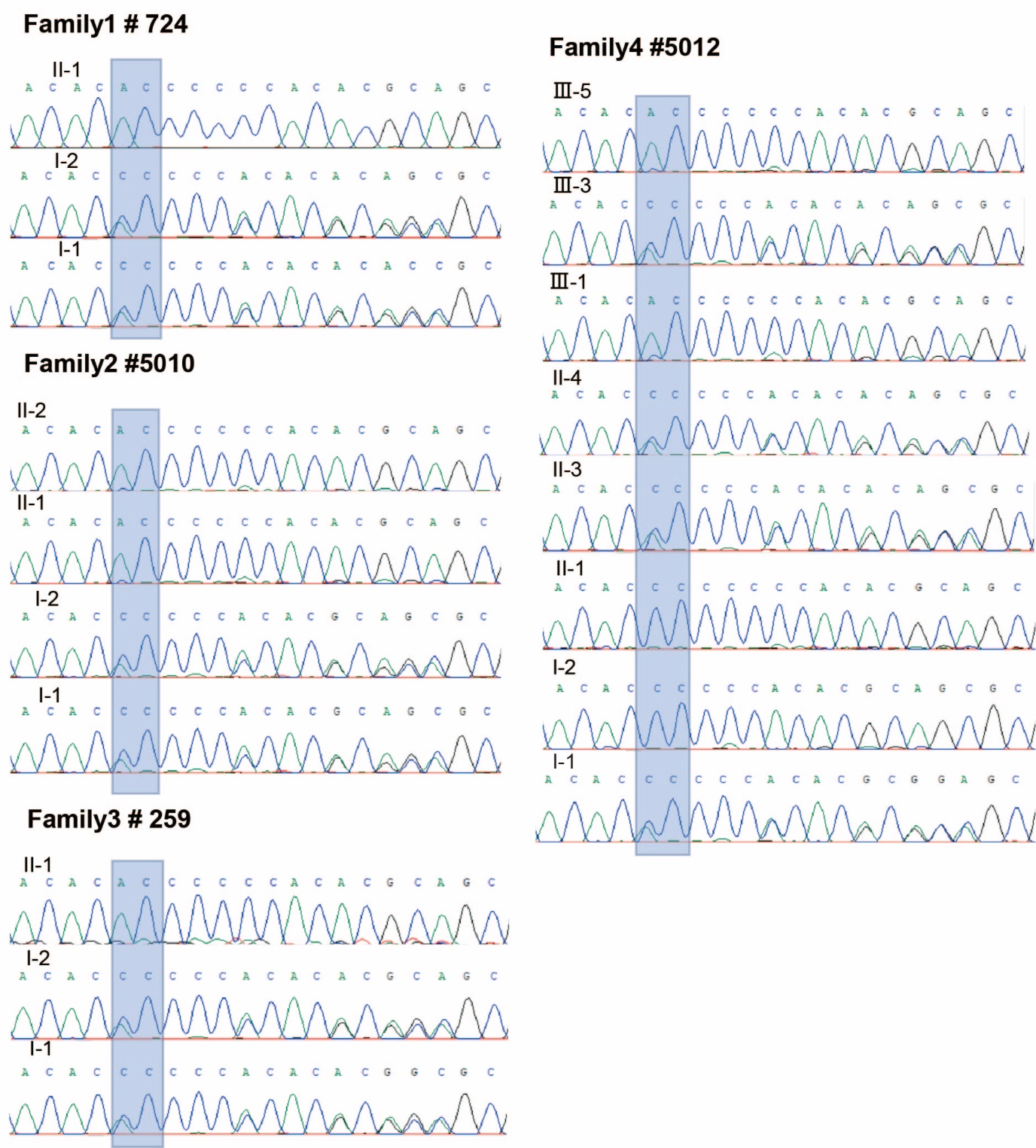


**Supplementary Figure 1** **Sanger sequencing of familial members.** These four CHD families showed the co-segregation of the homozygous variant and disease. The variant position is indicated by a blue square.


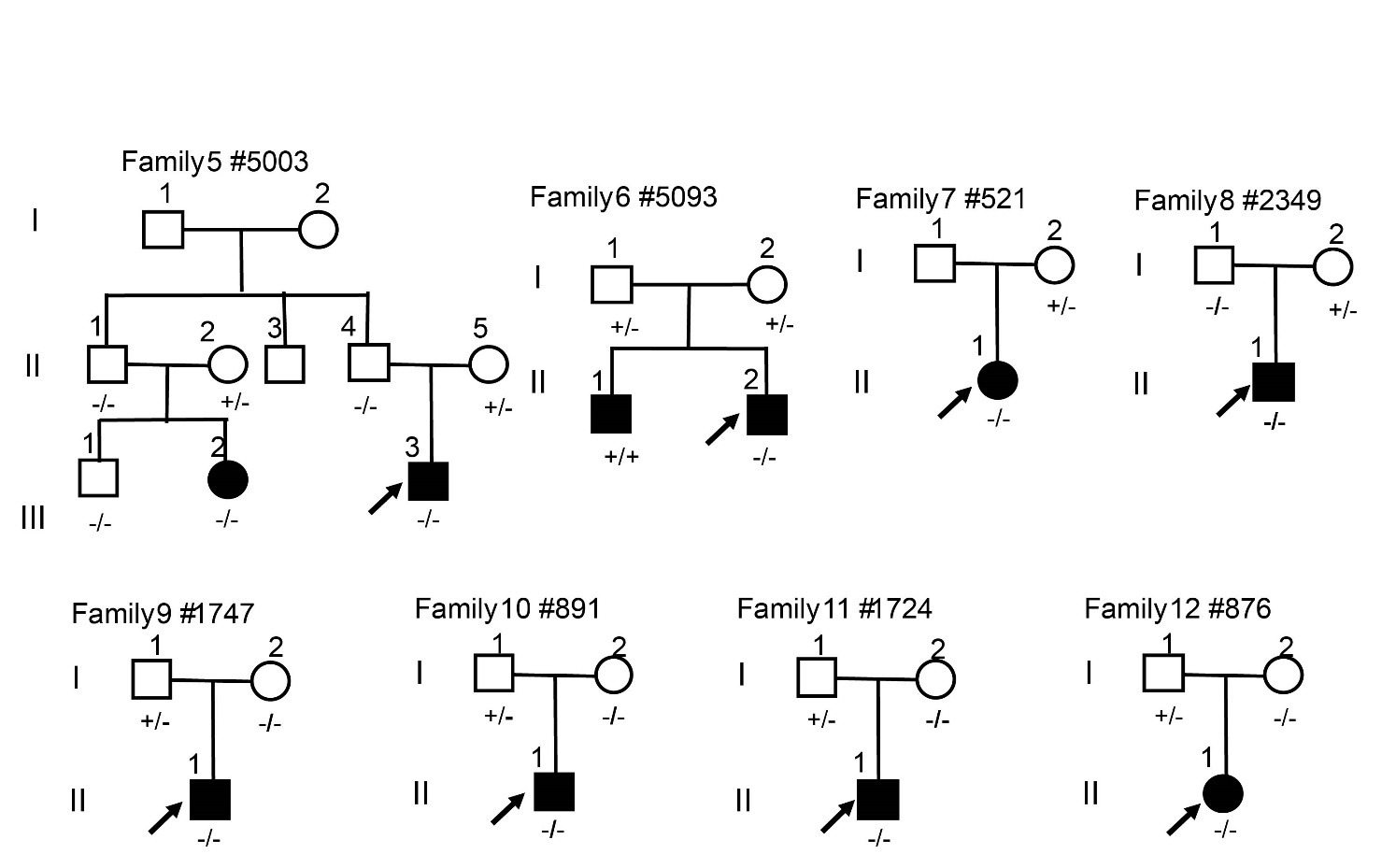


**Supplementary Figure 2** **Pedigree of the other 8 families.** The pedigree of families showing the phenotype and genotype of each family member; the arrow indicates the proband. Circles indicate female family members, and squares male family members. - denotes the c. -1279_-1278 insAC variant；+/- indicates individuals carrying a heterozygous variant; -/- indicates individuals with a homozygous variant.


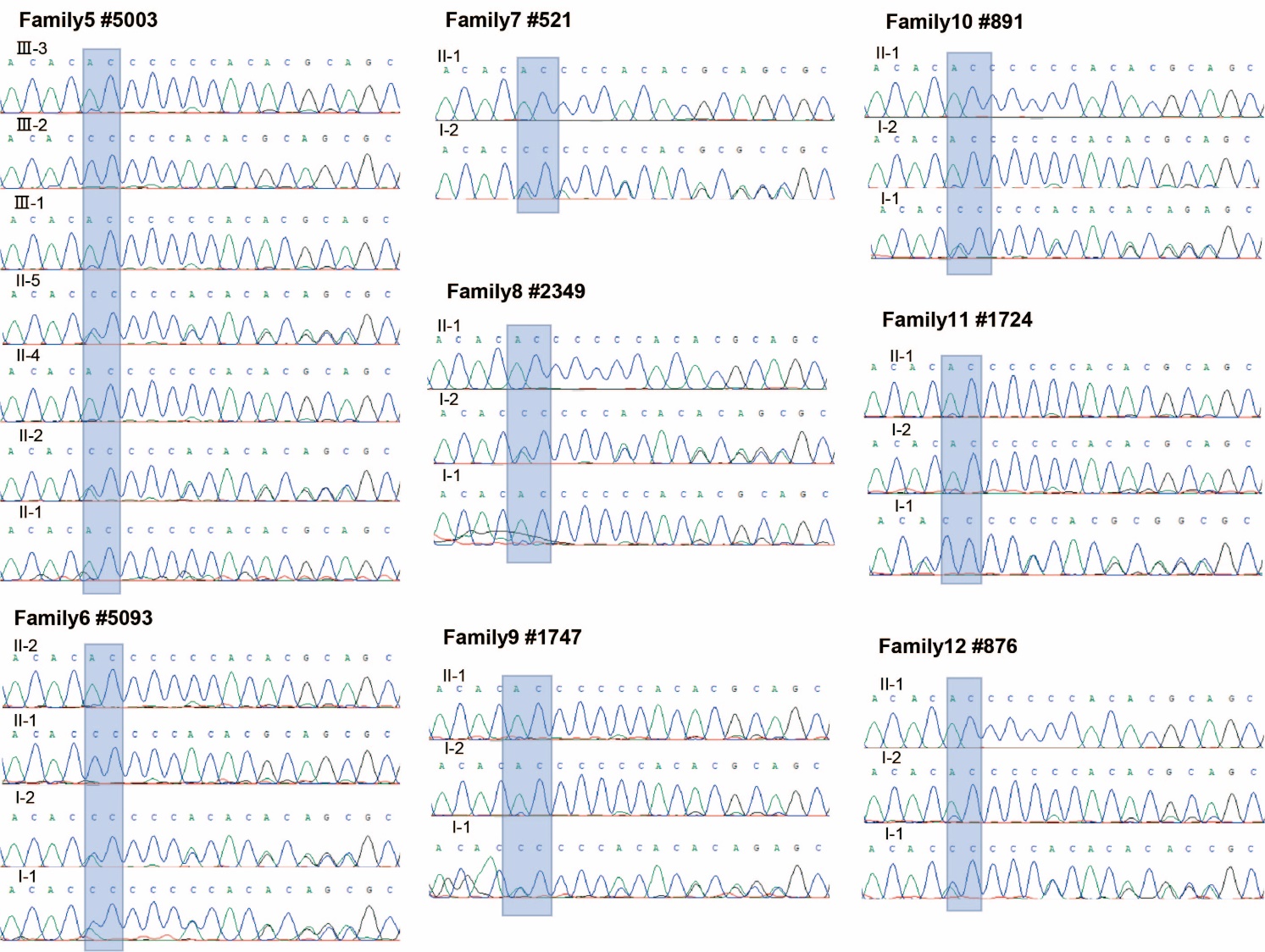


**Supplementary Figure 3 Sanger sequencing of familial members.** These 8 CHD families do not show homozygous variation and homo-segregation of disease. The variant position is indicated by a blue square.


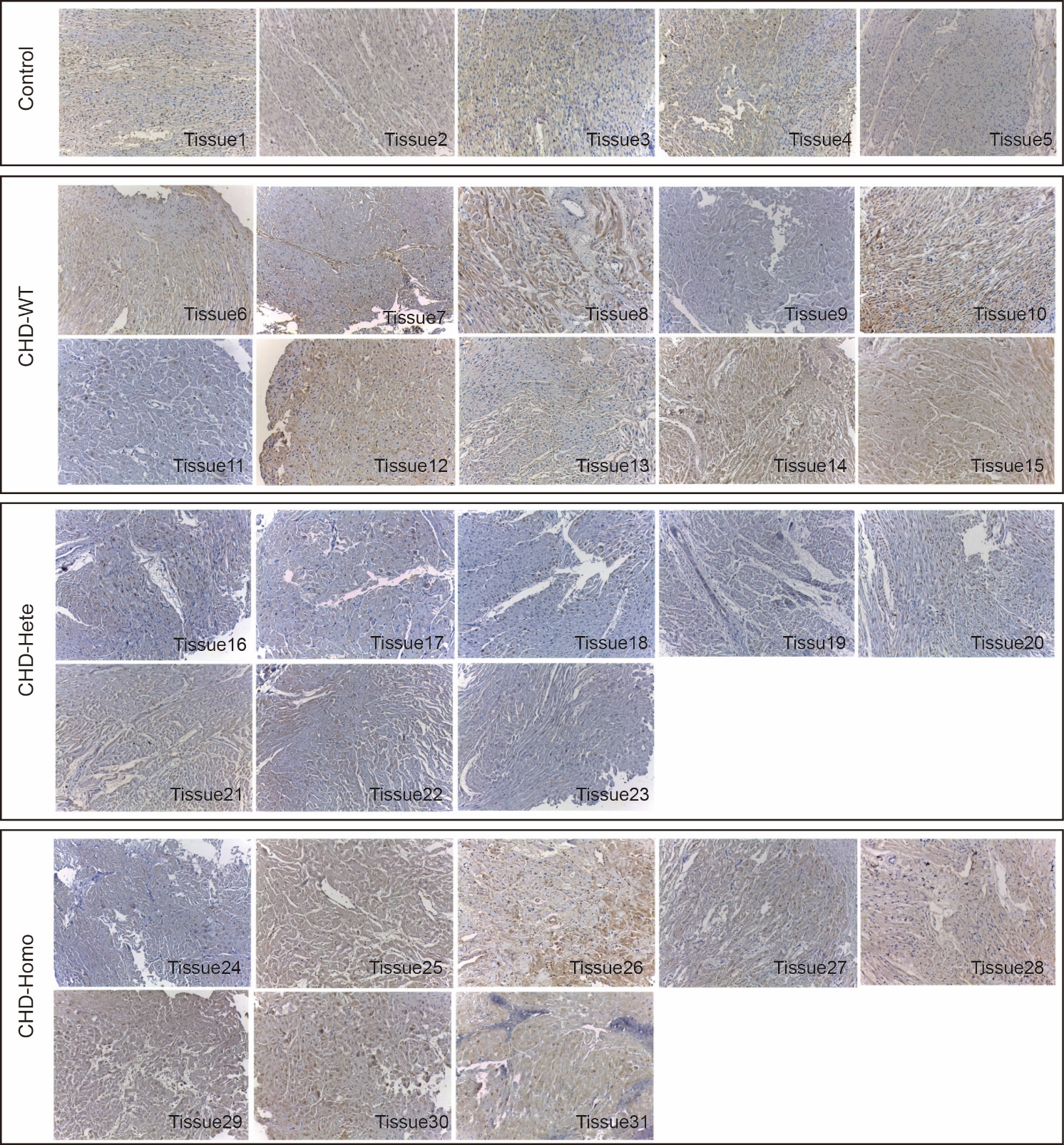


**Supplementary Figure 4 Immunohistochemical staining for HES1 in human RVOT from CHD patients and normal controls.** Representative RVOT images (20X) from 5 control cases (panel control), 10 CHD patients with wild-type genotype （panel CHD-WT）, 8 CHD patients with heterozygous variant (panel CHD-Hete), and 8 CHD patients with homozygous variant (panel CHD-Homo).


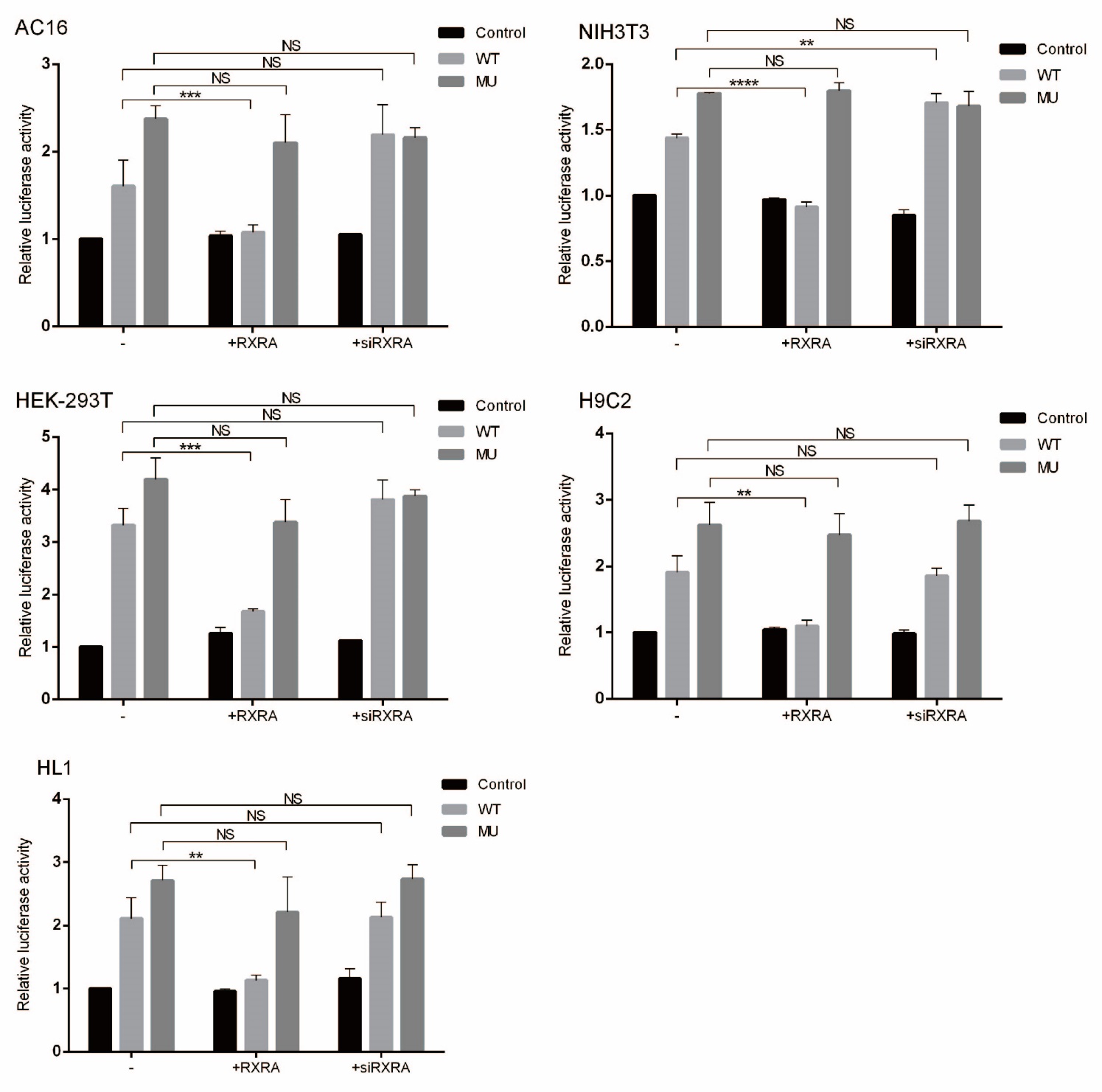


**Supplementary Figure 5 Variant affect the inhibitory effect of RXRA on enhancer activity. (displayed in Fig. 5).**
